# Alveolar soft part sarcoma in a young woman: A case report from Hong Kong

**DOI:** 10.1016/j.radcr.2022.02.076

**Published:** 2022-04-05

**Authors:** Max K.H. Yam, Kenneth K.K. Chan

**Affiliations:** Department of Radiology, North District Hospital, 9 Po Kin Road, Sheung shui, the New Territories, Hong Kong

**Keywords:** Alveolar soft part sarcoma, Sarcomas, Ultrasonography, Associations, Complications

## Abstract

Alveolar soft part sarcoma (ASPS) is a rare, highly vascular, deep soft tissue mesenchymal malignancy that is classically seen in the lower extremities of young adults. We reported a case of a 32-year-old young Chinese woman in Hong Kong with a biopsy-proven alveolar soft part sarcoma. The condition can be suggested by classical features using contrast-enhanced CT scans and MRIs or confirmed by image-guided biopsies. Early recognition of the condition is important due to its poor prognosis and lack of awareness. The mainstay of treatment for ASPS is complete surgical resection of the primary tumor and radiotherapy for microscopic residual disease at the primary site, or chemotherapy in special cases.

## Introduction

Alveolar soft part sarcoma (ASPS) is a rare, highly vascular, deep soft tissue mesenchymal malignancy. Typically, ASPS arises in muscles and deep soft tissue of the thigh or the leg (lower extremities), but can also appear in the upper extremities (hands, neck, and head).  Its prevalence is less than 1% of all primary soft-tissue sarcomas [1]. There is a slight female predilection in patients less than 30 years old [2]. The histogenic origin of ASPS remains uncertain although a myogenic or neural crest origin has been suggested [3].

Although ASPS displays a relatively indolent course, the ultimate prognosis is poor, and is often characterized by late metastases [4]. The mainstay of treatment for ASPS is complete surgical resection of the primary tumor and radiotherapy for microscopic residual disease at the primary site. In adults, ASPS is less effectively treated by standard chemotherapy but may still be considered in special cases [5]. The radiological features and treatment options of this disorder were discussed.

## Case report

A 32-year-old Chinese young woman was evaluated for right buttock pain for 2 weeks. She had good past health. The physical examination revealed a localized swelling and erythema over the right buttock. Her blood sample values were normal.

**Fig. 1:**
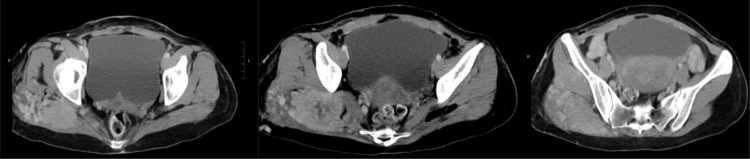
Contrast-enhanced CT scan of the pelvis. An irregular enhancing tumor centered at the right gluteus maximus muscle. Central hypoenhancement is noted suggestive of central necrosis. There are a few small enhancing nodules medial to the primary lesion (∼1 cm), likely representing satellite nodules. The tumor abuts the pyriformis medially and the gluteus medius muscle anteriorly. Right sciatic nerve is probably involved / in close proximity to the lesion. There is marked increase in neovascularity in the right gluteus maximus and medius muscles which are swollen, and in the subcutaneous tissue in the right buttock. Features suggestive of a highly vascular soft tissue sarcoma.

She then underwent a CT examination in which her scan revealed a large irregular enhancing tumor centered at the right gluteus maximus muscle. It was associated with hypertrophied intralesional and peritumoral vessels. Central hypoenhancement was suggestive of central necrosis. The lesion measured 6.8 cm x 5.3 cm x 8.0 cm in size. There were a few small enhancing nodules medial to the primary lesion (∼1 cm), those lesions likely represented satellite nodules. The tumor abutted the pyriformis medially and the gluteus medius muscle anteriorly. Right sciatic nerve was probably involved or in close proximity to the lesion. There was a marked increase in neovascularity in the right gluteus maximus and medius muscles which were swollen, and in the subcutaneous tissue in the right buttock. Features were suggestive of a highly vascular soft tissue tumor (Fig. 1).

**Fig. 2:**
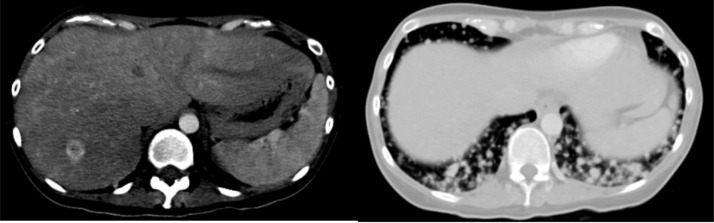
Contrast-enhanced CT scan of the abdomen and lung bases. (Right) There is an arterial enhancing nodule at segment VII of liver, suggestive of a vascular liver metastasis. (Left) Numerous lung nodules in included lung bases in keeping with extensive lung metastases.

There was an arterial enhancing nodule at the segment VII of liver (1.6 cm), suggestive of a vascular liver metastasis. Numerous lung nodules including lung bases were in keeping with extensive lung metastases. No pleural effusion was present. (Fig. 2).

**Fig. 3:**
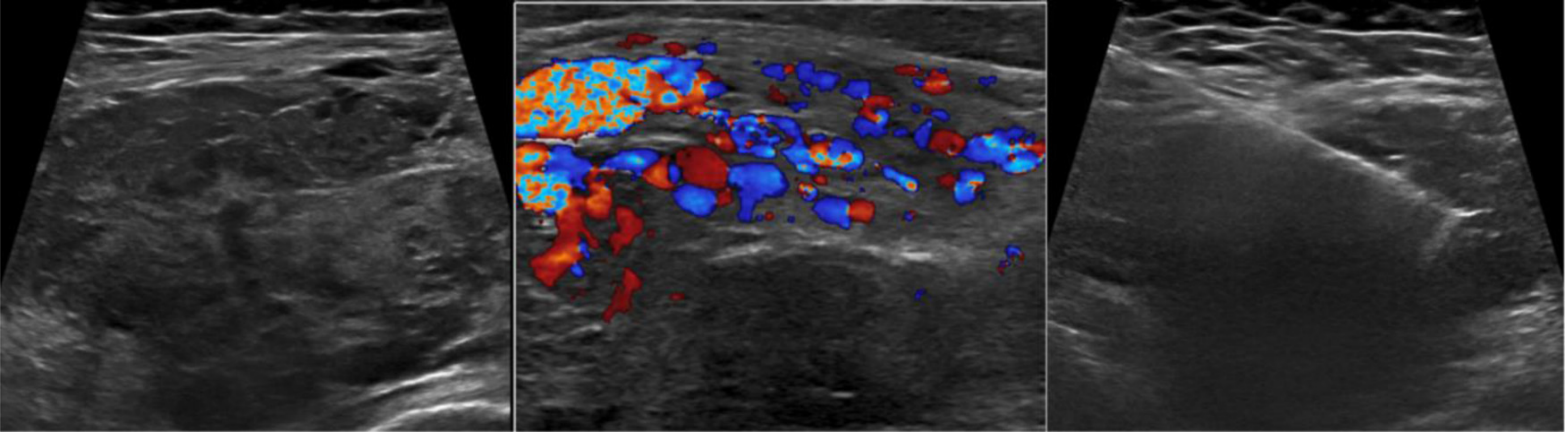
Ultrasound scan. (Left) There is a lobulated hypoechoic intramuscular mass at the right gluteal region. (Middle) The mass demonstrated pronounced vascularity within and around the lesion, associating with prominent vessels. (Right) Ultrasound guided biopsy of the right gluteal mass done.

The patient underwent an ultrasound investigation. The scan showed a highly vascularized soft tissue mass in the right gluteal region, associating with prominent vessels at the lateral subcutaneous aspect. It measured 7.5 × 3.4 × 7.3 cm in size.  Overall features were highly suspicious of malignancy in particular soft tissue sarcoma. Ultrasound guided biopsy was performed (Fig. 3).

**Fig. 4:**
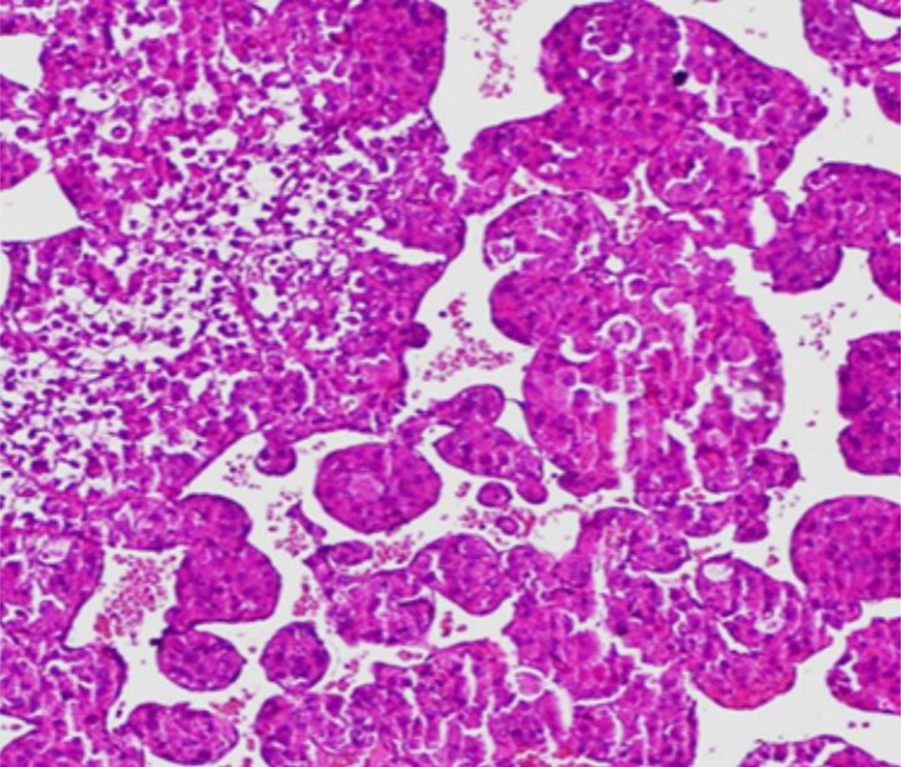
H&E stain. High power magnification. Nests of tumor cells forming pseudo-alveolar patterns and separated by thin sinusoidal vascular spaces. The tumor cells showed moderate to marked nuclear hyperchromasia and pleomorphism, inconspicuous to prominent nucleoli.

Pathology microscopic examination showed cores of tumor tissue composed of nests of tumor cells forming pseudo-alveolar patterns and separated by thin sinusoidal vascular spaces. The tumor cells showed moderate to marked nuclear hyperchromasia and pleomorphism, inconspicuous to prominent nucleoli, and granular eosinophilic cytoplasm. Mitosis was rare and focal necrosis was seen (Fig. 4).

**Fig. 5:**
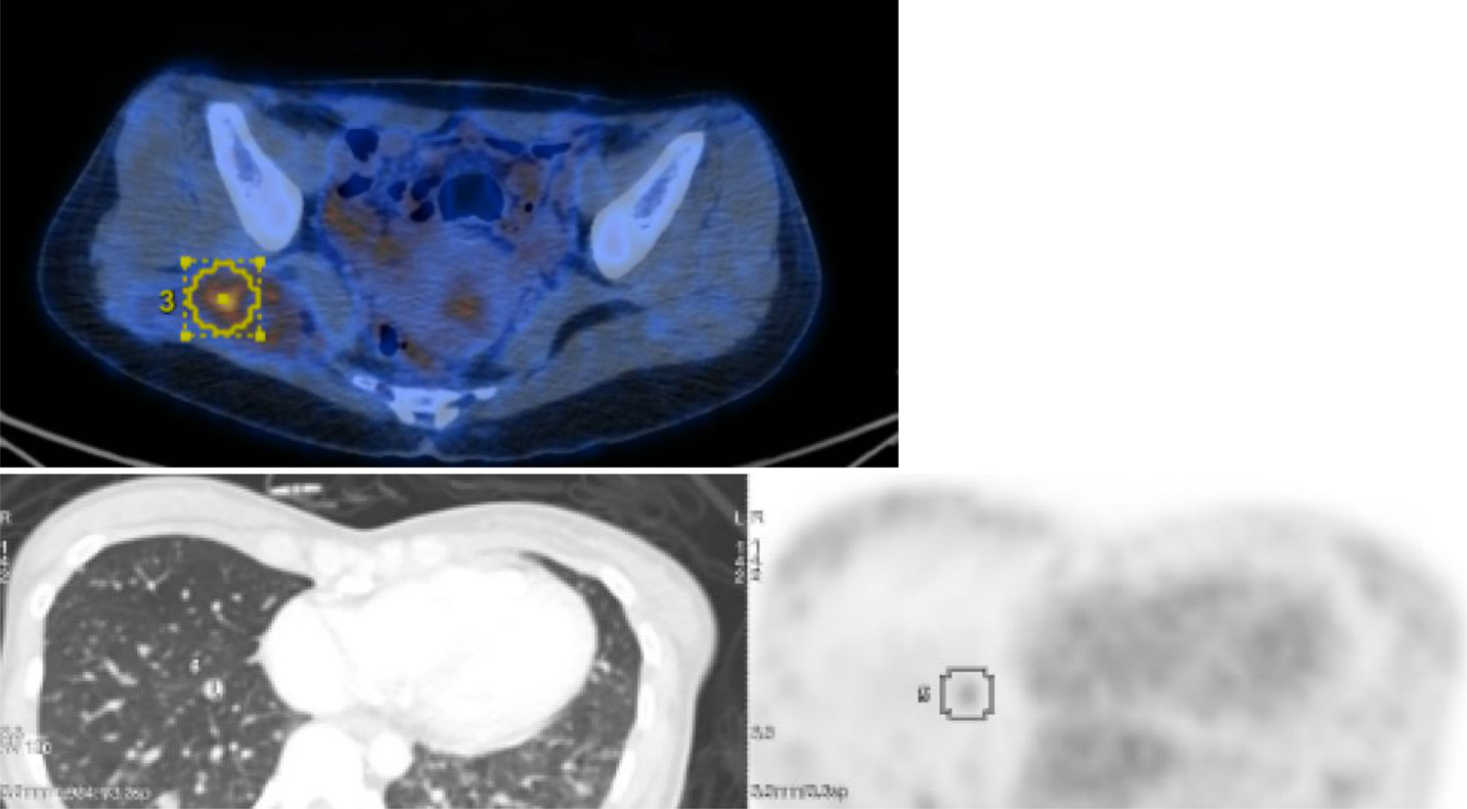
FDG 18 PET scan. The right gluteal soft tissue mass is moderately hypermetabolic (the upper image, SUVmax 4.6,  32 × 46 × 25 mm). Multiple non FDG avid or mildly hypermetabolic bilateral lung metastases are seen scattered randomly at both lungs, eg at medial right lower lobe ( the bottom image, SUVmax 1.5, 8 mm).

Subsequent F18-FDG positron emission tomography (PET) scan confirmed a hypermetabolic biopsy proven residual tumor at right gluteus maximus muscle with adjacent hypermetabolic tumorous satellite nodule, extensive non or mildly FDG avid bilateral lung metastases.  Mildly FDG avid lytic bony lesion at central S1 vertebral body, worrisome of residual bony metastasis. Non hypermetabolic enhancing nodule at segment VII of liver (Fig. 5). Ensuing diagnostic CT guided lung nodule biopsy confirmed metastatic nature of the mildly FDG avid lung nodule.

The patient refused any radiotherapy and after a multidisciplinary team meeting,  she was started on neoadjuvant chemotherapy instead,  consisting of pembrolizumab, and axitinib. The PET scan which was 5 months later showed metabolic response and interval reduction in the sizes of the primary tumor, neighboring satellite nodules, lung, and bone metastases. She was later arranged to have primary tumor resection.

## Discussions

Other differential diagnoses include undifferentiated pleomorphic sarcoma (UPS), previously known as malignant fibrous histiocytoma (MFH), is considered the most common type of soft tissue sarcoma [6]. However, the mean age of that diagnosis is 59 years and with a slight male predilection and has association with previous radiotherapy [7].

On the other hand, angiosarcomas frequently have metastases at the time of diagnosis, but are more common in males. Synovial sarcoma is a slow-growing and superficial tumor, without hypervascularity as a special feature. It often showed calcification, necrosis, and hemorrhage [8].

Alveolar soft part sarcoma (ASPS) is a rare, highly vascular, deep soft tissue mesenchymal malignancy that is classically seen in the lower extremities of young adults. They account for <1% of all soft tissue sarcomas. There is a slight female predilection in patients less than 30 years old.

Typically, ASPS is a slow-growing tumor, and often comes to clinical attention late in the disease process. About 65% of adults and 30% of children present with metastatic disease in the lungs, brain, bone, and/or lymph nodes. The prognosis of patients with ASPS is poor. In a large study by Lieberman, survival of 102 subjects dropped from 87% at 2 years to 18% at 20 years [9].

In adults, ASPS has a propensity for the lower extremities but can arise in unusual locations such as bone. In children, ASPS may arise in the head and neck, particularly the orbit and tongue. The diagnosis of ASPS relies on histopathology, immunohistochemistry, and molecular features. Histologically these tumors have a uniform pattern characterized by a pseudo alveolar or organoid arrangement of polygonal tumor cells separated by fibrovascular septa and delicate capillary-sized vascular channels . A characteristic unbalanced translocation, der(17)t(X:17) (p11;q25) results in the formation of an ASPL–TFE3 fusion protein. This translocation is seen almost exclusively in ASPS . An antibody directed against the C-terminus of the TEF3 protein is a highly sensitive and specific marker for ASPS. It has been suggested that the increased incidence of ASPS in females is related to their 2 X chromosomes, which doubles the probability of this translocation as compared to males.

The most consistent imaging feature of ASPS in one study was the presence of intra and peri-tumoral vessels which are large vessels at the superior and inferior poles that were evident by MRI and CT and seemed to converge toward the center of the tumor [10]. Additional features are the presence of small target lesions and a nodular internal architecture. Tumor margins tend to be lobulated and sharply defined, except in areas where vessels are present. When necrosis is present it is located centrally and surrounded by a thick rim of enhancing solid tumor.

These features are also consistent with others who reported the highly vascular nature of ASPS on imaging studies. The conventional angiographic findings of ASPS all describe similar findings on angiography. This hypervascular tumor demonstrates numerous enlarged vessels with arteriovenous shunting in the arterial phase followed by intense tumor staining [11].

The mainstay of treatment for ASPS is complete surgical resection of the primary tumor and radiotherapy for microscopic residual disease at the primary site. In adults, ASPS is less effectively treated by standard chemotherapy but may still be considered in special cases.

## Patient consent

Informed consent has been taken from the patient described in this case report.

Case reports and case series require informed consent, which should be documented in the paper. Studies on patients or volunteers require ethics committee approval and informed consent, which should be documented in the paper. Appropriate consents, permissions and releases must be obtained where an author wishes to include case details or other personal information or images of patients and any other individuals in an Elsevier publication. Written consents must be retained by the author but copies should not be provided to the journal. Only if specifically requested by the journal in exceptional circumstances (for example if a legal issue arises) the author must provide copies of the consents or evidence that such consents have been obtained. For more information, please review the Elsevier Policy on the Use of Images or Personal Information of Patients or other Individuals. Unless you have written permission from the patient (or, where applicable, the next of kin), the personal details of any patient included in any part of the article, and in any supplementary materials (including all illustrations and videos) must be removed before submission.
